# Comparison of Modified Lake’s Extender for Cryopreservation of Hungarian Giant and Chrabrany Rabbit Sperm

**DOI:** 10.3390/ani16213473

**Published:** 2026-11-03

**Authors:** Éva Kissné Váradi, Jakub Vozaf, Jaromír Vašíček, Jozef Berčik, Andrej Baláži, Peter Chrenek, Árpád Drobnyák, Zsuzsa Szabó, Éva Török, Krisztina Liptói, Barbara Végi

**Affiliations:** 1 Institute for Farm Animal Gene Conservation, National Centre for Biodiversity and Gene Conservation, Isaszegi Road 200, H-2100 Gödöllő, Hungary; drobnyak.arpad@nbgk.hu (Á.D.); szabo.zsuzsa@nbgk.hu (Z.S.); torok.eva@nbgk.hu (É.T.); liptoi.krisztina@nbgk.hu (K.L.); vegi.barbara@nbgk.hu (B.V.); 2 Division of Animal Genetics and Reproduction, Department of Animal Production, National Agricultural and Food Centre (NPPC), Hlohovecká 2, 95141 Lužianky, Slovakia; jakub.vozaf@nppc.sk (J.V.); jaromir.vasicek@nppc.sk (J.V.); andrej.balazi@nppc.sk (A.B.); peter.chrenek@nppc.sk (P.C.); 3 Institute of Biotechnology, Faculty of Biotechnology and Food Science, Slovak University of Agriculture in Nitra, Tr. A, Hlinku 2, 94976 Nitra, Slovakia; xbercikj@uniag.sk

**Keywords:** native rabbit breeds, cryotolerance, modified Lake’s extender

## Abstract

Sperm cryopreservation is the most effective method for the long-term preservation of genetic resources. However, rabbit sperm cryopreservation remains challenging. One of the key factors influencing freezing success is the choice of semen diluent; however, no specific diluent has been established for rabbit semen. In this study, we compared the effectiveness of modified Lake’s diluent and evaluated the cryosurvival of semen from the native Hungarian breed and a native Slovak rabbit breed. Both breeds were subjected to the same static freezing procedure. The following parameters were evaluated: motility, viability, plasma membrane integrity, mitochondrial activity, acrosomal status, and intracellular reactive oxygen species. In fresh samples, viability (93.5% vs. 77.4%), total motility (84.6% vs. 67.0%), and progressive motility (76.1% vs. 47.0%) were significantly higher for the Hungarian Giant than in the Chrabrany rabbit samples. Following cryopreservation, viability (56.4% vs. 43.4%), total (39.2% vs. 20.5%) and progressive (20.6% vs. 8.5%) motility, acrosomal status (72.2% vs. 44.9%) and ROS activity (33.8% vs. 24.6%) were higher in samples from the Chrabrany breed. The results suggest that the modified Lake’s diluent may also be suitable for other rabbit breeds and highlight the importance of developing breed-specific cryopreservation protocols.

## Simple Summary

Although artificial insemination with fresh, diluted semen has long been standard practice in rabbit breeding, the use of cryopreserved semen remains challenging, mainly because of the low efficiency of semen cryopreservation. One of the key factors affecting the success of cryopreservation is the selection of a suitable semen diluent. In this study, the cryopreservation efficiency of semen from two native rabbit breeds, the Hungarian Giant and the Chrabrany (Slovak), was investigated using the same Lake’s diluent, modified for rabbit semen. This diluent had previously provided the best cryopreservation results for the Hungarian Giant. The same static freezing procedure was applied to semen from both breeds. Sperm motility, viability, plasma membrane integrity, mitochondrial activity, acrosomal status, and intracellular reactive oxygen species levels were evaluated. Whereas fresh semen quality was higher in the Hungarian Giant, most measured parameters were higher for Chrabrany rabbit samples after freezing and thawing. This result suggests that Chrabrany rabbit sperm tolerated freezing and thawing better and that the modified Lake’s diluent may also be useful for preserving semen from other rabbit breeds.

## 1. Introduction

Cryopreservation studies of rabbit semen have not been conducted for as long as those in other livestock species. While the use of fresh, diluted semen for artificial insemination has become widespread, sperm cryopreservation has mainly been used for experimental and gene conservation purposes. Although several studies have reviewed the practical conditions for rabbit sperm freezing [1,2], there are still many gaps in this area.

First, there is currently no semen extender specifically developed for rabbit sperm cryopreservation. The Tris-based diluents initially used for this purpose were subsequently replaced mainly by French commercial extenders (Cortalap, Lepus, INRA 82 and INRA 96), while more recently, extenders originally developed for the cryopreservation of stallion semen, such as INRAFreeze and BotuCrio, have become increasingly prominent. BotuCrio extender, which contains egg yolk, has been successfully used in numerous studies for rabbit sperm freezing [3,4,5]. However, in our own comparative study, the presence of sterilized egg yolk made microscopic evaluation difficult, particularly when using computer-assisted sperm analysis techniques [6]. Egg yolk of animal origin also poses a hygienic disadvantage and carries a risk a bacterial contamination [7]. Furthermore, egg yolk is difficult to standardize because its composition can vary considerably [8]. Due to these disadvantages, increasing efforts have been made to develop and use egg yolk-free extenders for rabbit sperm freezing. A fundamental requirement for a semen extender is to provide sufficient nutrients and protective substances during semen processing. In the case of cryopreservation, it should protect spermatozoa from freezing- and thawing-induced damage through as many mechanisms as possible. Since rabbit spermatozoa generally require extenders with lower osmolality and pH [9], we previously prepared a modified Lake’s extender for this purpose.

The Lake’s extender, originally developed for the dilution of domestic rooster semen, was modified by reducing the amount of fructose. This modification resulted in lower osmolality, making the extender suitable for use in rabbits [10]. The modified Lake’s extender was supplemented with glycerol (1%) and dimethylformamide (DMF) (4%) as cryoprotectants. In addition to contributing to the maintenance of low osmolality, glycerol, which penetrates the sperm membrane more slowly than some other permeating cryoprotectants, can provide dual protection when used in combination with another permeating cryoprotectant. The addition of two permeating CPAs, usually dimethyl sulfoxide (DMSO) or other amides together with glycerol, has been a common practice in rabbit sperm cryopreservation [11,12,13,14,15]. When used in combination, glycerol should be applied with caution, as its concentration should not exceed 1.5% when used together with DMSO [16]. Although the DMSO–glycerol combination is the most commonly used, other combinations have also been tested. The combination of 1 M acetamide with glycerol (0, 2 or 5%) in the extender did not improve the survival rate after sperm freezing [17]. In contrast, Domingo et al. [3] reported acceptable motility results following cryopreservation using 1% glycerol and 4% methylformamide. Based on these positive results, we tested the protective effect of glycerol in combination with DMF when using the modified Lake’s extender, as DMSO alone had not proven sufficiently effective in our preliminary studies [10].

Numerous reviews have been published on the effectiveness of various sperm freezing protocols [1,2]; however, their findings are difficult to evaluate because they involve numerous variables, including different extenders, CPAs, breeds and other methodological factors. Following the evaluation of different cryopreservation methods, including extenders and cryoprotectants, an additional challenge emerged: similar to other animal species, the freezability of semen varies widely among rabbit breeds and lines. Differences in semen quality in both fresh and frozen/thawed semen have been reported among Slovak indigenous rabbit breeds [18], in agreement with the findings of Mocé et al. [19], who reported that sperm parameters are affected by genotype and breed. If differences have already been observed among Slovak breeds, differences in semen quality and freezing tolerance may also exist among native rabbit breeds from different countries. To the best of our knowledge, apart from the experiment described above, the freezability of semen from different rabbit breeds has not yet been directly compared. Such studies are warranted because differences in freezing tolerance between breeds have been reported [20], suggesting that each breed may require an individual freezing protocol. The combined effects of individual and breed-related differences support the view that variations in sperm freezability are, to some extent, genetically determined [21].

Based on the above findings, the present study aimed to evaluate the applicability of the modified Lake’s extender for the cryopreservation of semen from the Slovak native Chrabrany rabbit breed and to compare semen freezability between the Hungarian Giant, Hungary’s only native rabbit breed, and the Chrabrany. These breeds were selected because of their relevance to the conservation of local rabbit genetic resources within our collaborative project and the availability of biological material. The study provides an initial assessment of the applicability of the same cryopreservation approach to two native rabbit breeds.

## 2. Materials and Methods

### 2.1. Animals and Semen Collection

The study was conducted simultaneously at the National Centre for Biodiversity and Gene Conservation, Institute for Farm Animal Gene Conservation in Hungary and at the National Agricultural and Food Centre, Division of Animal Genetics and Reproduction in Slovakia.

**Figure 1:**
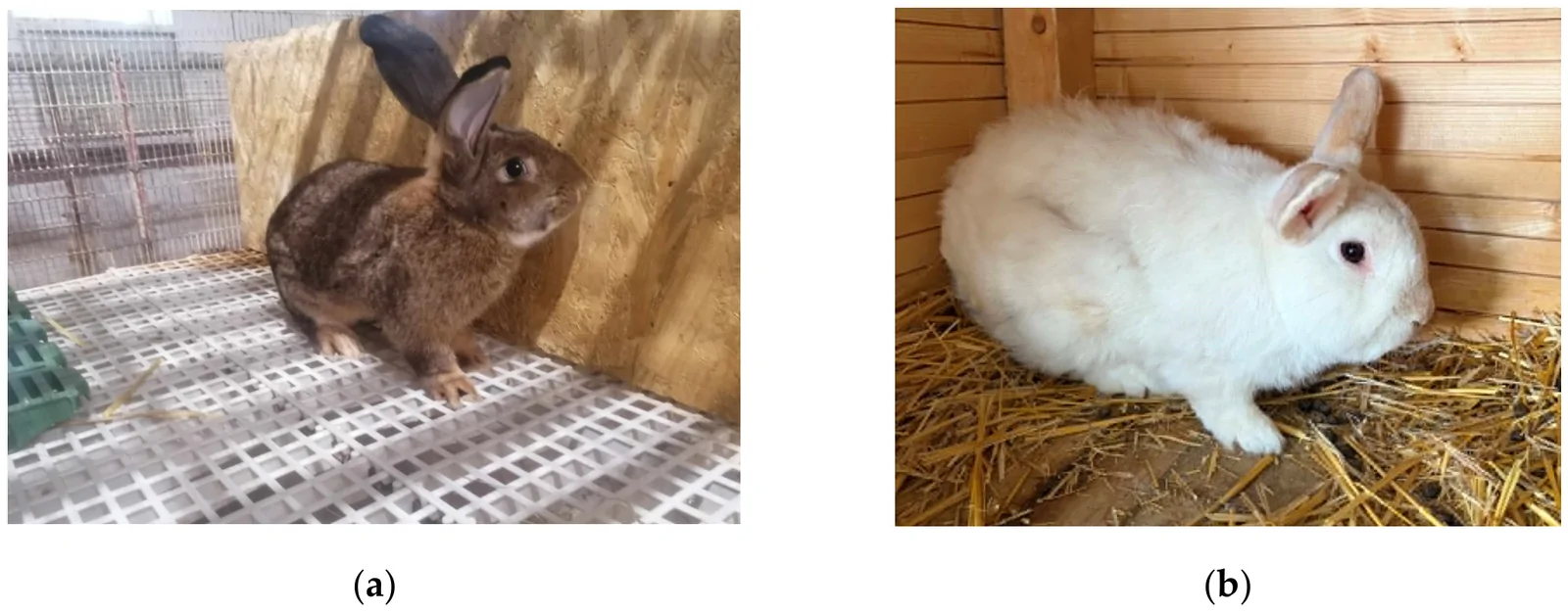
(**a**) Hungarian Giant, (**b**) Chrabrany rabbit.

Ten sexually mature Hungarian Giant bucks, aged 10–22 months, average body weight 6.65 kg (6.12–6.87 kg), were housed individually (Figure 1) under 12L:12D photoperiod at an ambient temperature of approximately 18–20 °C. The animals were fed 150–200 g/day of a special rabbit breeding diet prepared according to an institutional recipe (17.38% crude protein, 3.04% crude fat, 16.79% crude fibre, 8.78% crude ashes, 10.35AME/MJ metabolizable energy). Hay and drinking water supplemented with probiotics and vitamins (Gastroferm M-vit, Premium Product Ltd., Kiskunmajsa, Hungary) were provided *ad libitum*.

Five sexually mature bucks of the Chrabrany rabbit breed, aged 2–3 years, average body weight 4.83 kg (4.5–5.1 kg), were used (Figure 1). The rabbits were housed individually under controlled environmental conditions at an ambient temperature of approximately 15–18 °C and a 12L:12D photoperiod. The animals had *ad libitum* access to water and were fed a standard commercial rabbit diet (min. 16.5% crude protein, 16% crude fibre, 3% fat, 14–16% starch, 9–11 MJ/kg metabolizable energy).

Semen was collected once weekly from each of the 10 Hungarian Giant and 5 Chrabrany bucks using a preheated artificial vagina (IMV Technologies, L’Aigle, France). Semen volume and motility were assessed for both ejaculates, and the gel plug was removed when present. Motility was assessed subjectively using a light microscope (Leitz Diaplan; Leica Microsystems GmbH, Wetzlar, Germany) equipped with a 20× objective. Sperm motility was scored on a scale of 0 to 5 based on the proportion of spermatozoa exhibiting progressive motility, as follows: 0, no movement; 1, 5–20%; 2, 20–40%; 3, 40–60%; 4, 60–80%; and 5, more than 80%. When assessing rabbit sperm motility, the presence of the gel plug in the semen sample, even after its removal, must be taken into consideration, as it may hinder the precise distinction between these categories. Based on subjective motility scoring on a scale of 0–5, only samples of adequate quality (score 4–5) were pooled and used for cryopreservation.

### 2.2. Cryopreservation Protocol

The freezing experiments were performed in 10 replicates from Hungarian Giant and 7 replicates from Chrabrany, based on pooled samples, owing to differences in successful sperm donation between animals. The number of bucks providing semen and the total number of ejaculates included in the pooled samples are presented in Table 1.

The same semen freezing procedure was used for both breeds. The previously developed modified Lake’s extender for rabbit sperm (0.96 g sodium glutamate, 0.07 g magnesium-acetate, 0.5 g potassium acetate, 0.3 g polyvinylpyrrolidone, 0.4 g D-Fructose in 100 mL distilled water; pH: 6.95; 300 mOsm/kg) was used, supplemented with 1% glycerol and 4% dimethylformamide (DMF).

**Figure 2:**
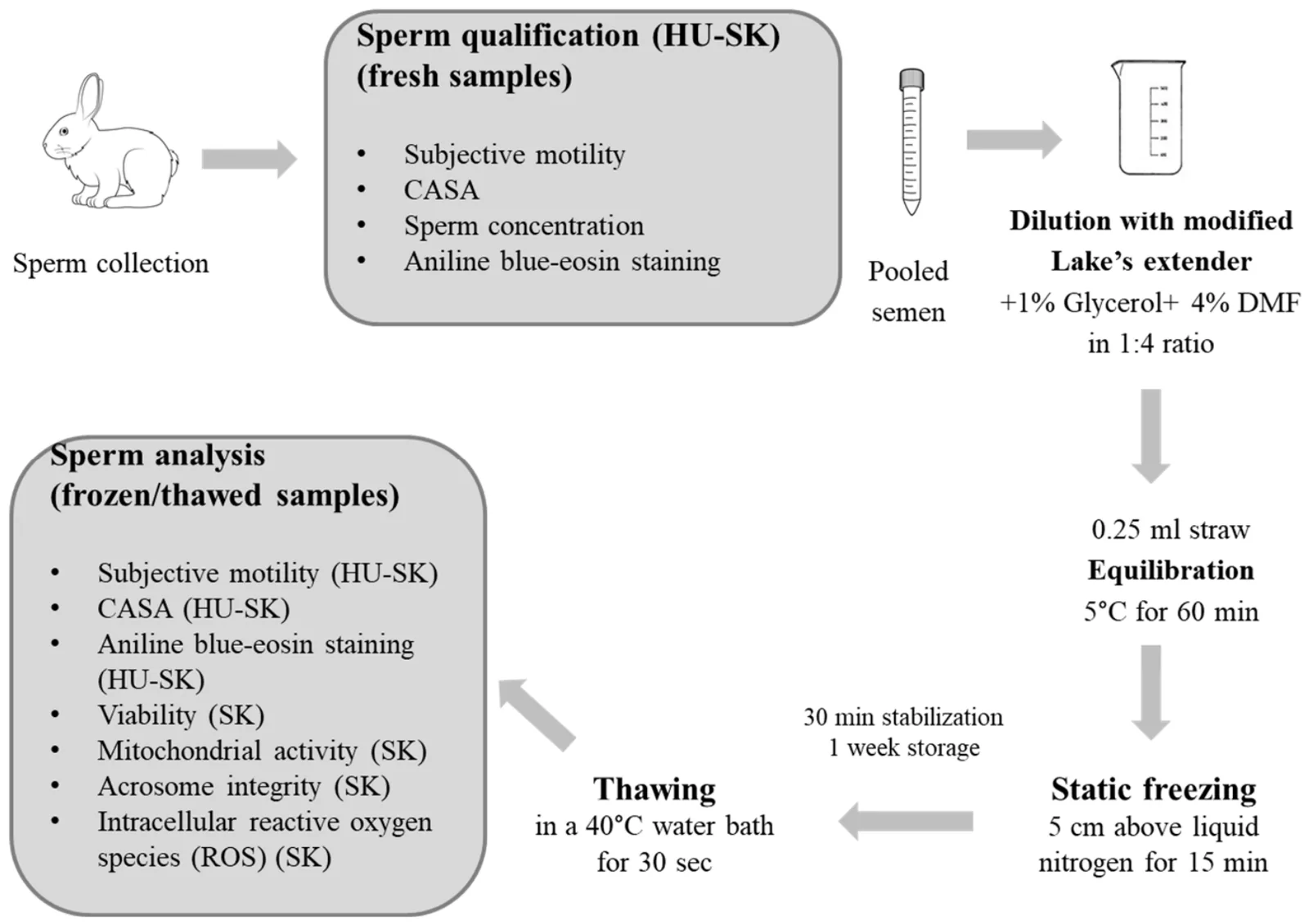
Freezing protocol used for Hungarian Giant and Chrabrany semen. (HU-SK): Analysis examined in Hungarian and Slovak laboratory. (SK): Analysis examined only in Slovak laboratory.

Pooled semen samples were diluted at a ratio of 1:4 with the modified Lake’s extender containing glycerol and DMF. The samples were loaded into 0.25-mL straws at final sperm concentrations ranging from 23.7 to 52.4 × 10^6^ spermatozoa/mL for the Hungarian Giant rabbits and from 25 to 40 × 10^6^ spermatozoa/mL for the Chrabrany rabbits. The straws were then equilibrated at 5 °C for 60 min. Following equilibration, the straws were placed 5 cm above the surface of liquid nitrogen for 15 min. Both laboratories used the same freezing unit designed for 20 straws (Minitube GmbH, Tiefenbach, Germany). After freezing, the straws were kept in the freezing box for a 30-min stabilization period. They were then transferred to a liquid nitrogen storage tank and stored in liquid nitrogen for 1 week before thawing. Thawing was performed in a water bath at 40 °C for 30 s (Figure 2).

### 2.3. Semen Evaluations

#### 2.3.1. Sperm Motility and Concentration

At the Hungarian laboratory, sperm motility in Hungarian Giant semen was assessed using a computer-assisted sperm analysis system (CASA, Microptic S.L. SCA^®^, Barcelona, Spain). For both fresh and frozen/thawed semen, samples were diluted with modified Lake’s extender at a 1:12 (vol/vol) ratio. A 10 µL aliquot of diluted semen was loaded onto a prewarmed microscope slide, and multiple fields were examined until at least 500 spermatozoa had been analyzed using a 10× objective. Sperm concentration was determined using a Makler counting chamber (Sefi-Medical Instruments Ltd., Haifa, Israel).

At the Slovakian laboratory, motility of fresh and frozen/thawed Chrabrany semen was assessed using SpermVision^TM^ (MiniTube, Tiefenbach, Germany) at 200× magnification. A 10 µL drop of semen diluted at a 1:10 (vol/vol) ratio was placed in a Makler counting chamber (Sefi-Medical Instruments Ltd., Haifa, Israel), and motility was assessed as described above. Sperm concentration was also determined using SpermVision^TM^ software (Version 3.8).

The following motility parameters were assessed: total motility (TM; sperm velocity > 5 µm s^−1^), and progressive motility (PM; sperm velocity > 20 µm s^−1^).

Although the two laboratories used different software systems for motility assessment and there were some methodological differences, frozen/thawed Hungarian Giant samples were analyzed in both laboratories using both methods. No differences were observed between motility values obtained in the two laboratories. Therefore, the data obtained using the two methods were considered comparable, including for fresh semen.

#### 2.3.2. Viability Assessment Using Aniline Blue–Eosin Staining

**Figure 3:**
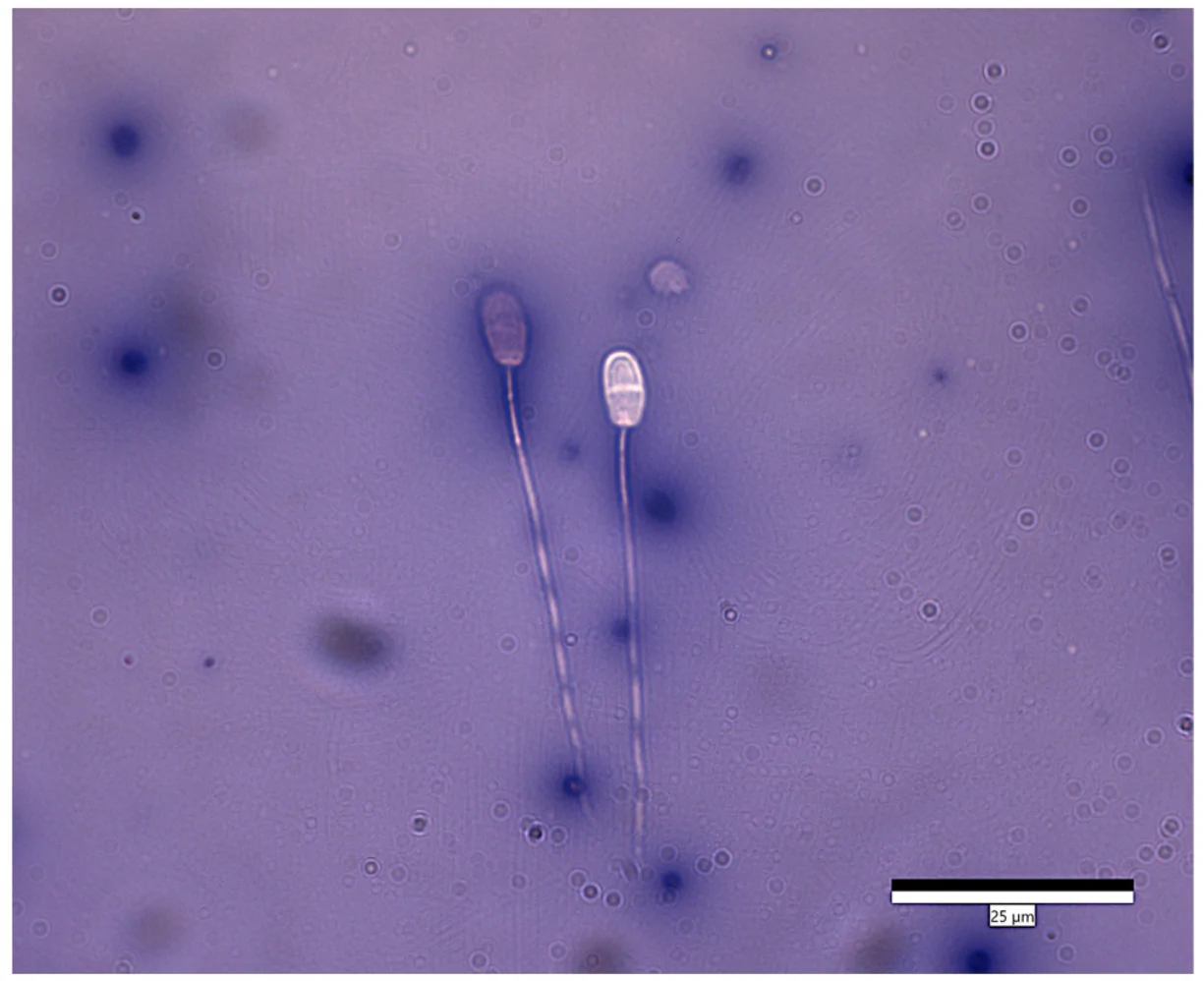
Aniline blue–eosin vital staining. Live spermatozoa: white colour, dead spermatozoa: dark colour.

To determine the proportion of live and dead spermatozoa, both laboratories used the same aniline blue–eosin vital staining procedure for fresh and frozen/thawed samples. This method is based on eosin penetration into spermatozoa with damaged plasma membranes, resulting in pink staining of the spermatozoa, whereas aniline blue provides a uniform dark background (Figure 3). To prepare the aniline blue–eosin stain (Aniline SIGMA-Aldrich, Eosin Y Merck, Budapest, Hungary), 0.2 g of eosin and 0.8 g of aniline blue were dissolved in 10 mL of modified Lake’s extender. A 20 µL aliquot of the stain was pipetted into an Eppendorf tube, followed by 10 µL of sperm, and the contents were gently mixed. Subsequently, 10 µL of the mixture was placed onto a microscope slide to prepare a smear, which was fixed with warm air. The smears were examined under oil immersion at 1200× magnification. A total of 200 cells were evaluated per smear [22].

#### 2.3.3. Sperm Analyses by Flow Cytometry

**Figure 4:**
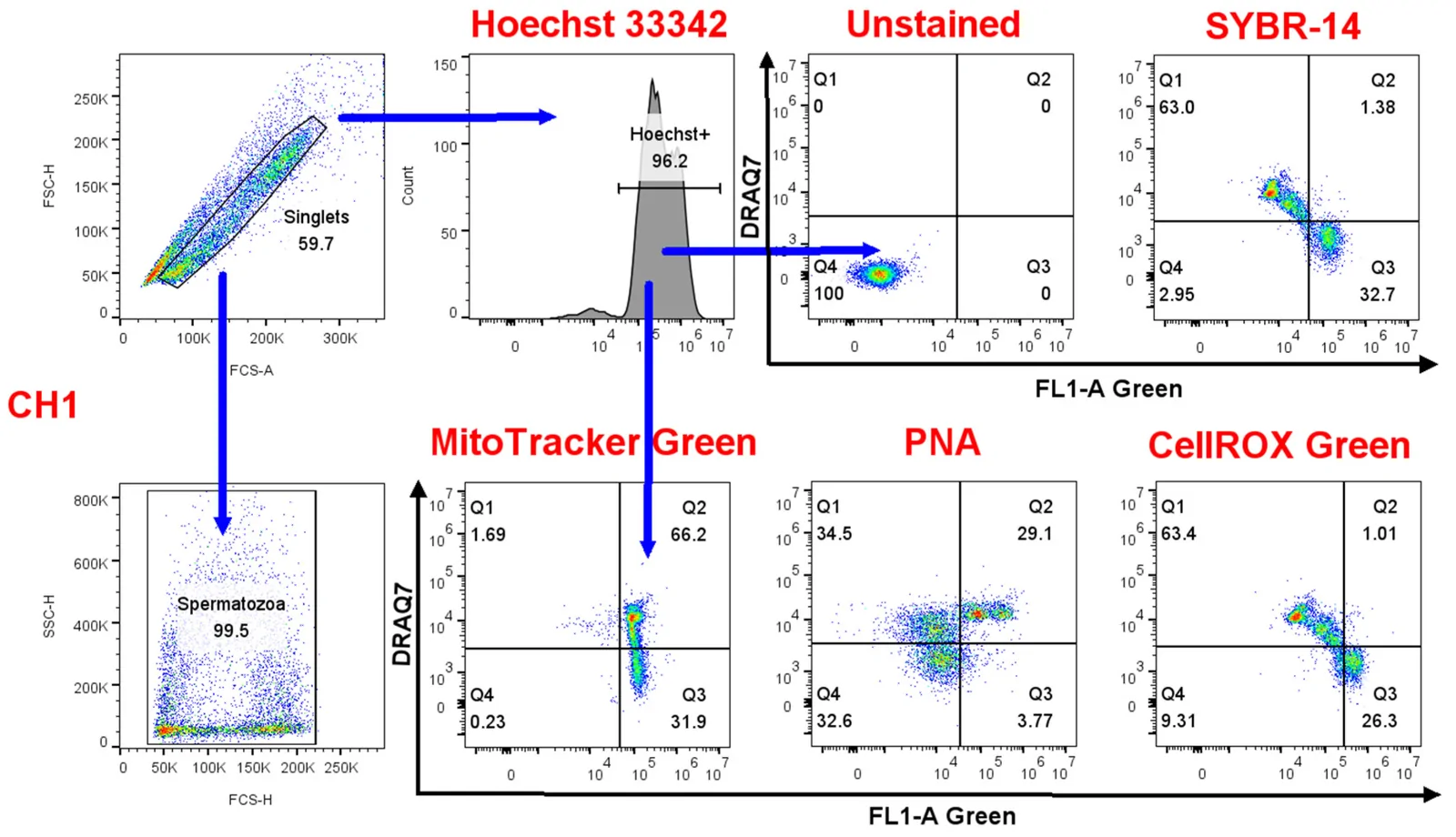
Illustrative dot plots used for the flow cytometric evaluation of rabbit spermatozoa. Spermatozoa were first gated based on singlets to exclude debris and cellular aggregates, as shown in the lower-left dot plot (FSC/SSC). Subsequently, events containing DNA stained with Hoechst 33342 were gated, as shown in the upper-middle dot plot. This gating strategy was used to distinguish unstained spermatozoa (control) from spermatozoa stained with different fluorescent probes to assess cell viability (live cells: Q3, SYBR-14^+^/DRAQ7^−^; dead cells: Q1, SYBR-14^−^/DRAQ7^+^, and Q2, SYBR-14^+^/DRAQ7^+^), mitochondrial activity (high mitochondrial activity: Q3, MitoTracker Green^+^/DRAQ7^−^), acrosomal status (PNA-positive spermatozoa: Q2, PNA^+^/DRAQ7^+^, and Q3, PNA^+^/DRAQ7^−^), and reactive oxygen species (ROS) production (CellROX-positive spermatozoa: Q2, CellROX^+^/DRAQ7^+^, and Q3, CellROX^+^/DRAQ7^−^).

The following analyses were performed exclusively on frozen/thawed samples, as only the laboratory in Slovakia was equipped with a flow cytometer. Flow cytometric evaluation of sperm function was performed according to the methodologies described by Vašíček et al. [23] and Kuželová et al. [24], with modifications to optimize the protocol for rabbit spermatozoa and Hoechst 33342-positive spermatozoa gating (Figure 4).

Samples were analyzed immediately after the staining procedure using a CytoFLEX S (Beckman Coulter, Inc., Brea, CA, USA) equipped with near-UV (375 nm, 50 mW), violet (405 nm, 70 mW), blue (488 nm, 50 mW) and red (638 nm, 40 mW) lasers. Fluorescence emission was detected through three optical channels: FL1 (525/40 nm; green fluorescence), FL6 (660/10 nm; far-red fluorescence for DRAQ7), and FL12 (450/45 nm; blue fluorescence for Hoechst 33342). For each sample, a minimum of 10,000 spermatozoa were acquired at low flow rate (600–1000 events/s) using logarithmic signal amplification. Sperm populations were identified using sequential gating strategies, including singlet and Hoechst 33342 gating, to exclude debris, non-sperm particles, and cellular aggregates, thereby ensuring the analysis of morphologically intact single spermatozoa. Data acquisition was performed using CytExpert 2.6 software (Beckman Coulter, Inc., Brea, CA, USA; 2011–2023), whereas subsequent data processing and quantitative analyses were performed with FlowJo™ software (v10.10.0; BD Biosciences, San Jose, CA, USA).

Sperm plasma membrane integrity was measured using SYBR-14 green fluorescent (LIVE/DEAD^®^ Sperm Viability Kit, Thermo Fisher Scientific Inc., Waltham, MA, USA) and DRAQ7 far-red nucleic acid fluorescent dyes (BioStatus Limited, Shepshed, UK). Briefly, a semen sample containing 1 × 10^6^ spermatozoa was first incubated with SYBR-14 for 10 min at 37 °C in the dark. DRAQ7 was then added without washing, followed by a further 10 min incubation at room temperature. During flow cytometric analysis, without washing, spermatozoa with intact plasma membranes were identified as SYBR-14^+^/DRAQ7^−^, whereas dead or membrane-compromised spermatozoa were identified as SYBR-14^+^/DRAQ7^+^ and SYBR-14^−^/DRAQ7^+^, respectively.

Mitochondrial activity was detected using MitoTracker^®^ Green FM (Thermo Fisher Scientific Inc., Waltham, MA, USA) and DRAQ7 far-red fluorescent dyes. A semen sample containing 1 × 10^6^ spermatozoa diluted in 500 µL PBS was incubated with MitoTracker^®^ Green for 10 min at 37 °C in the dark. Following centrifugation at 500× *g* for 5 min at room temperature, the sample was stained with DRAQ7 and analyzed by flow cytometry. High mitochondrial activity was identified in live spermatozoa positive for MitoTracker^®^ Green (MitoTracker^®^ green^+^/DRAQ7^−^).

Acrosomal status was assessed using peanut agglutinin (PNA) conjugated with Alexa Fluor 488 (Thermo Fisher Scientific Inc., Waltham, MA, USA). A semen sample containing 1 × 10^6^ spermatozoa diluted in 200 µL PBS was incubated with PNA for 15 min at room temperature in the dark. Following centrifugation at 500× *g* for 5 min at room temperature, the samples were washed with PBS and stained with DRAQ7. During flow cytometric analysis, PNA-positive spermatozoa were classified as acrosome-damaged, including both PNA^+^/DRAQ7^−^ and PNA^+^/DRAQ7^+^ populations.

Intracellular reactive oxygen species (ROS) were detected using CellROX Green fluorescent stain (Thermo Fisher Scientific Inc., Waltham, MA, USA). A semen sample containing 1 × 10^6^ spermatozoa diluted in 500 µL PBS was incubated with CellROX for 15 min at 37 °C in the dark. Following incubation, the sample was washed and stained with DRAQ7 and analyzed by flow cytometry as previously described. ROS-positive cells were classified as CellROX^+^/DRAQ7^−^ and CellROX^+^/DRAQ7^+^.

The final working concentrations of all fluorescent dyes used in the study are reported in the previous study of Vašíček and Kuželová et al. [23,24].

### 2.4. Statistical Analysis

Statistical analyses were performed using StataNow 19.5 software (StataCorp LLC, College Station, TX, USA). For data expressed as percentages, arcsine transformation was applied prior to statistical analysis [25]. Normality was assessed using the Shapiro–Wilk test. All evaluated parameters except acrosomal status followed a normal distribution. Normally distributed data were analyzed using one-way analysis of variance (ANOVA), followed by Tukey’s post hoc test. For non-normally distributed data, the Mann–Whitney U test was used to compare the two groups. Multivariate analysis of variance (MANOVA) was used to assess the breed × treatment interaction.

## 3. Results

### 3.1. Fresh Sperm Quality of the Breeds

According to the sperm viability assessment, the proportion of live spermatozoa in fresh samples was significantly higher in the Hungarian Giant than in the Chrabrany breed.

In the CASA analysis, both total and progressive motility were significantly higher in Hungarian Giant samples compared with Chrabrany semen (Table 2). Thus, the initial quality of fresh semen, based on the proportion of live spermatozoa, total and progressive motility, was significantly higher for the Hungarian Giant than the Chrabrany breed. No detectable difference was found in the sperm concentration between the two breeds, although the two testing methods differed.

Following equilibration at 5 °C for 60 min, subjective sperm motility was 4.4, while viability assessed by aniline blue–eosin staining was 94.65% live spermatozoa in the Hungarian Giant breed. These findings suggest that no marked deterioration in subjective sperm motility or viability occurred during the equilibration period. These assessments were performed only in the Hungarian Giant breed; therefore, corresponding data were not available for comparison with the Chrabrany breed.

### 3.2. Sperm Quality After Freezing/Thawing

In contrast to the fresh samples, the proportion of live spermatozoa after freezing/thawing was significantly higher in the Chrabrany than in the Hungarian Giant breed. Both total and progressive motility were also significantly higher in Chrabrany samples than in Hungarian Giant semen (Table 3).

**Figure 5:**
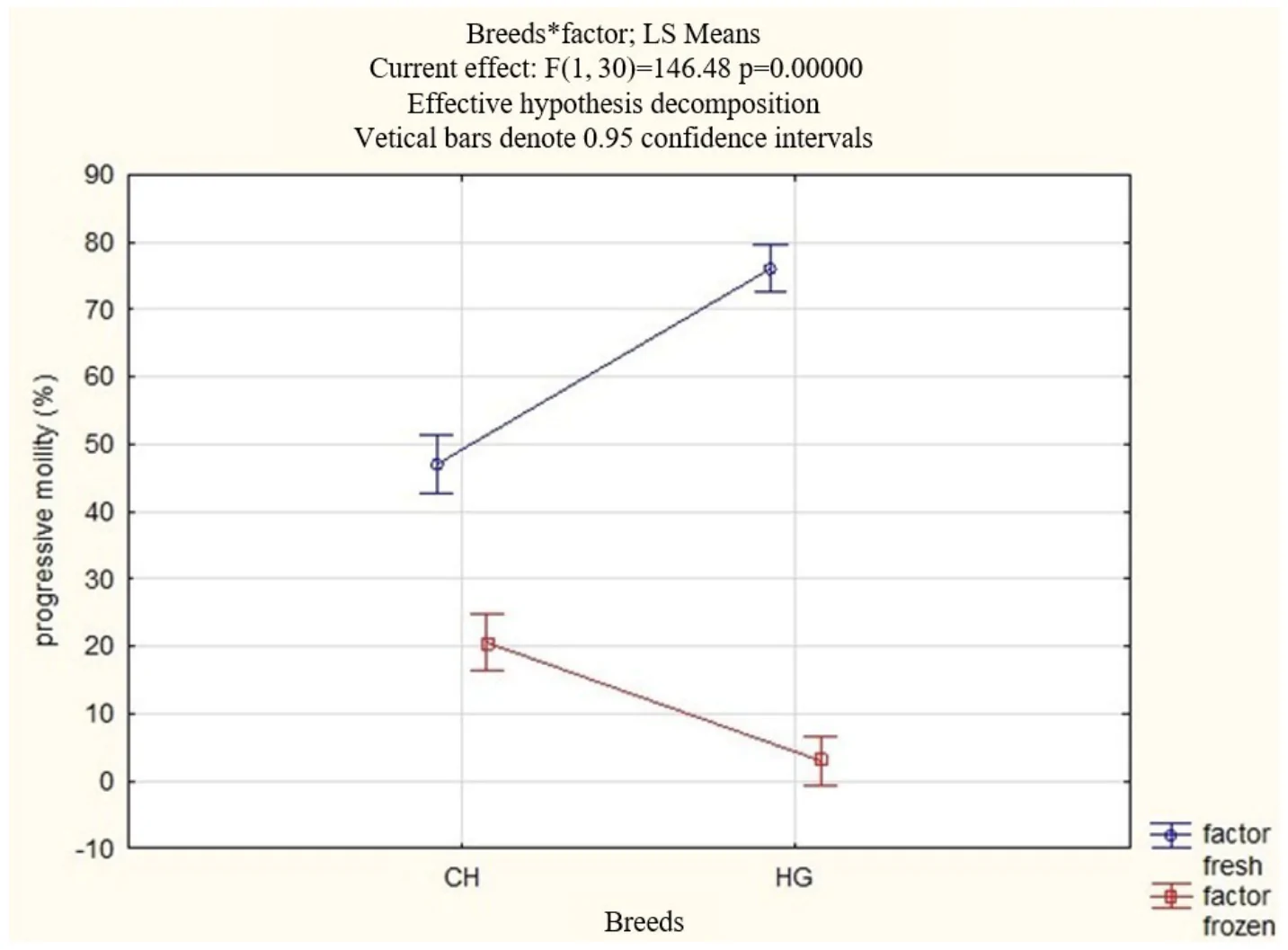
Progressive sperm motility (%) in fresh and frozen/thawed semen from Chrabrany (CH) and Hungarian Giant (HG) rabbit breeds. Data are presented as least-squares means (LS means) with 95% confidence intervals. A significant breed * treatment interaction was observed (F(1, 30) = 146.48, *p* < 0.001).

**Figure 6:**
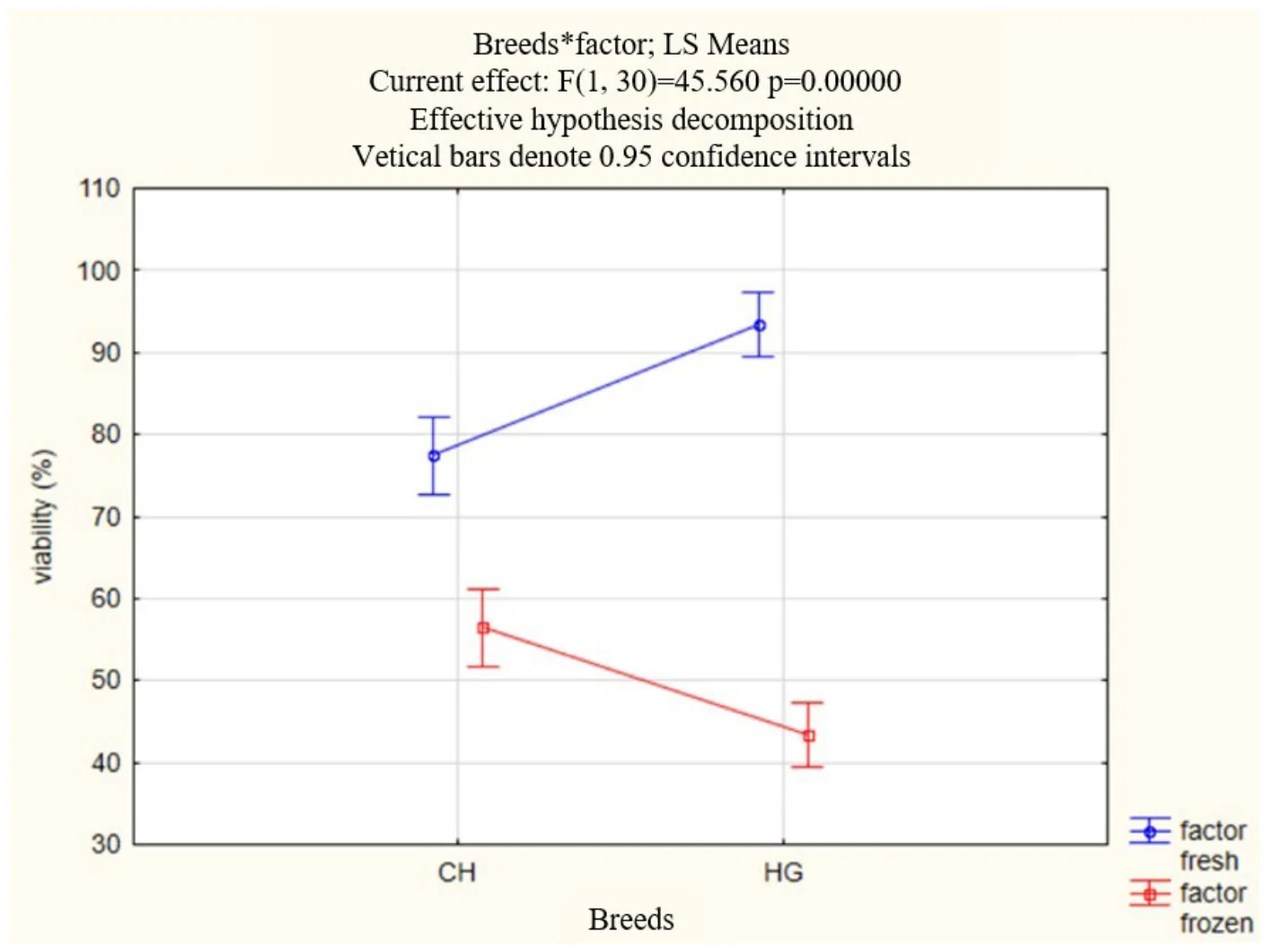
Viability (%) in fresh and frozen/thawed semen from Chrabrany (CH) and Hungarian Giant (HG) rabbit breeds. Data are presented as least-squares means (LS means) with 95% confidence intervals. A significant breed * treatment interaction was observed (F(1, 30) = 45.560, *p* < 0.001).

Factorial analysis of variance showed that breed, treatment, and the interaction between breed and treatment all had significant effects on the sperm parameters examined (Figure 5 and Figure 6). The significant interaction indicates that the effect of treatment differed between breeds; therefore, the main effects should be interpreted in the context of this interaction.

A significant breed × treatment interaction was observed for progressive motility (F(1, 30) = 146.48, *p* < 0.00001). Fresh semen from Hungarian Giant (HG) showed higher progressive motility than Chrabrany (CH) semen (approximately 76% vs. 47%, respectively). Cryopreservation markedly reduced progressive motility in both breeds, but the reduction was substantially greater in Hungarian Giant samples, where progressive motility decreased to 8.5%, compared with 20.6% in Chrabrany semen. Thus, the effect of cryopreservation on progressive motility differed markedly between breeds.

A significant breed × treatment interaction was also observed for sperm viability (F(1, 30) = 45.56, *p* < 0.00001). In fresh semen, viability was higher in Hungarian Giant than in Chrabrany sperm (approximately 93% vs. 77%, respectively). Following cryopreservation, viability decreased in both breeds; however, the reduction was substantially greater in Hungarian Giant sperm, resulting in lower post-thaw viability compared with Chrabrany sperm (approximately 43% vs. 56%, respectively). Similar to progressive motility, the effect of cryopreservation on sperm viability differed between the breeds.

Flow cytometric analysis of frozen/thawed samples showed no significant difference between the breeds in sperm plasma membrane integrity or in the proportion of dead/membrane-compromised spermatozoa. Mitochondrial activity was also similar between the breeds.

In contrast, acrosome damage was significantly higher in Hungarian Giant than in Chrabrany semen. Furthermore, intracellular reactive oxygen species (ROS) levels were significantly higher in frozen/thawed Hungarian Giant semen than in Chrabrany semen (Table 4).

## 4. Discussion

In addition to damage caused by the freezing procedure itself, including heat shock, ice crystal formation, and ROS production, rabbit semen cryopreservation is further complicated by breed- and individual-related variability in freezing tolerance. Therefore, breed-specific cryopreservation protocols may be required, with the selection of an effective semen extender representing an important first step. A comprehensive study by Kubovicova et al. [2] reflects the semen extenders most commonly used in recent years. However, comparing the effectiveness of different extenders is difficult because different cryoprotectants are used in combination with different extenders. Furthermore, the diluents have been tested in different rabbit breeds. Among commercial extenders, BotuCrio has become increasingly popular and has been successfully used for sperm freezing in Californian and New Zealand hybrids, achieving total motility of approximately 50% [3,4,5], although it has so far been used only rarely with native breeds [10]. In our previous study, the effectiveness of BotuCrio, INRAFreeze and the modified Lake’s extender was compared in the Hungarian Giant breed. The Lake’s extender was modified by reducing the amount of fructose, which resulted in lower osmolality and made the extender suitable for use in rabbits. The use of the modified Lake’s extender supplemented with dimethyl sulfoxide resulted in no differences in semen quality after freezing/thawing among the extenders tested. When DMSO was replaced with glycerol (1%) and DMF (4%), the modified Lake’s extender resulted in a higher proportion of live, morphologically normal spermatozoa and higher total motility than BotuCrio [10]. Since the modified Lake’s extender proved most effective for the Hungarian Giant in the previous experiment, we aimed to test its suitability for another native breed as well. Therefore, in the present study, sperm freezing efficiency was compared between the Hungarian Giant and Chrabrany native rabbit breeds using the same modified Lake’s extender and freezing procedure, based on conventional in vitro sperm quality assessments complemented by flow cytometric analyses.

It is well known that significant differences in semen quality exist among rabbit breeds, and that spermatological parameters can differ according to breed and genotype [19]. Based on this, the first step was to compare the quality of fresh semen between the two breeds. The initial sperm quality, assessed based on the proportion of live spermatozoa, total and progressively motility, was significantly lower in Chrabrany than in Hungarian Giant semen, indicating a clear difference between the examined breeds. This finding is consistent with the earlier observations of Kuliková et al. [18], who reported variability in fresh and frozen/thawed semen quality among Slovak native breeds. After cryopreservation, however, the pattern was reversed. Each of the parameters mentioned above was significantly higher in Chrabrany than Hungarian Giant semen. Total motility was nearly twice as high in Chrabrany semen as in Hungarian Giant semen (39.2 vs. 20.5%), and progressive motility was also substantially higher in Chrabrany semen (20.6 vs. 8.5%). Interestingly, although Hungarian Giant spermatozoa exhibited higher progressive motility in the fresh state, this apparent advantage was not retained after cryopreservation. The pronounced reduction in progressive motility in Hungarian Giant semen, despite a substantial proportion of live spermatozoa remaining after thawing, suggests that cryopreservation may impair sperm functionality beyond the loss of plasma membrane integrity. This indicates that spermatozoa from Chrabrany breed tolerate cryopreservation better than those from the Hungarian Giant. Furthermore, as observed in our previous study, semen with highest initial quality is not necessarily the most resistant to cryopreservation.

The relatively low post-thaw motility observed in the present study is in line with findings reported for several native rabbit breeds. Cryopreservation of Mexican Chinchilla rabbit semen resulted in only 9.4% progressive motility [26]. Cryopreservation of Bianca Italiana semen resulted in 40% total motility but only 20% progressive motility [27], similar to the values reported for the Slovak Pastel rex breed (32 TM%, 16 PM%) [18]. In contrast, higher motility values were observed in New Zealand White lines for both total (45%) and progressive (29%) motility [4].

To investigate possible causes of the low progressive motility and assess the extent of cellular damage, flow cytometric analyses of frozen/thawed semen were performed. The examined parameters included viability analyses, mitochondrial activity, acrosomal status, and intracellular reactive oxygen species (ROS) formation. Among the measured parameters, acrosome damage (72 vs. 45%) and ROS formation (34 vs. 25%) were significantly higher in Hungarian Giant than in Chrabrany semen. In comparison with commercial hybrids such as New Zealand White, lower levels of acrosome damage (17.4%) and ROS production (25%) were reported after static freezing using BotuCrio extender [4]. These differences may suggest that native breeds are more sensitive to cryopreservation, which may be related to their greater individual variability, whereas hybrids may be more uniform [28]. Following cryopreservation, both acrosome damage and ROS production may increase from approximately 10–15% to around 40% [29]. High levels of ROS in semen can cause oxidative stress, which may directly damage spermatozoa, induce DNA fragmentation and apoptosis, and impair sperm motility [30,31]. These detrimental effects may also contribute to the lower functional quality observed in frozen/thawed Hungarian Giant semen.

Taken together, these findings indicate that the two breeds differed not only in their initial semen quality, but also in their response to cryopreservation. The different responses of the two breeds to cryopreservation cannot be explained solely by sperm survival. In Hungarian Giant semen, the marked reduction in progressive motility despite the relatively high proportion of viable spermatozoa suggests that the functional capacity of surviving spermatozoa maybe substantially impaired by freezing. The higher levels of acrosome damage and ROS formation observed in Hungarian Giant semen further support the possibility that cryopreservation-induced cellular and functional damage contributed to the pronounced reduction in post-thaw motility.

We acknowledge that the relatively low post-thaw motility and viability may limit the practical application of the current protocol and that its fertilizing capacity remains to be confirmed through artificial insemination trials. However, the value of semen cryopreservation should also be considered in the context of genetic resource conservation. In this context, the primary objective is not necessarily to maximize reproductive efficiency or economic profitability, but rather to preserve genetic diversity and maintain the potential for future breed recovery. Thus, even a protocol resulting in suboptimal post-thaw sperm quality may contribute to the conservation of endangered or numerically small native breeds, provided that its reproductive effectiveness can ultimately be demonstrated [32].

## 5. Conclusions

Overall, the results indicate that the modified Lake’s extender may also be suitable for the cryopreservation of semen from other rabbit breeds. At the same time, the present study revealed differences in freezing tolerance the two examined breeds, highlighting the importance of developing breed-specific cryopreservation protocols. Further studies, including artificial insemination trials, are required to validate the fertilizing capacity of cryopreserved semen and determine the practical applicability of the proposed protocol.

## Figures and Tables

**Table 1:** Number of bucks providing semen and total number of ejaculates included in each replicate.

	Replicates	Number of Sperm Donor Bucks	Total Number of Ejaculates	Total Number of Ejaculates Excluded	Total Number of Ejaculates Used in Pooled Samples	Average Number of Individual Ejaculates/Each Pooled Samples
Hungarian Giant	10	10	88	10	78	7.8
Chrabrany	7	5	35	4	31	6.2

**Table 2:** Percentage of live spermatozoa, concentration, and total and progressive motility of fresh semen in Chrabrany and Hungarian Giant rabbit breeds. Values are presented as mean ± standard error of the mean (SEM) for normally distributed variables. (Lowercase letters a-b indicate significant differences between the rows with in the same column, * *p* < 0.05, ** *p* < 0.01).

Fresh, Diluted Samples	Aniline Blue–Eosin Staining	Concentration	CASA Analysis	
	Live Spermatozoa (%)	Million/mL	Total Motility (%)	Progressive Motility (%)
Chrabrany	77.4 ± 1.9 ^a^*	700 ± 50.7	67 ± 1.9 ^a^**	47 ± 2.9 ^a^**
Hungarian Giant	93.5 ± 0.34 ^b^	701 ± 65.6	84.6 ± 1.4 ^b^	76.1 ± 1.7 ^b^

**Table 3:** Percentage of live spermatozoa, total and progressive motility of frozen/thawed semen in Chrabrany and Hungarian Giant rabbit breeds. Values are presented as mean ± standard error of the mean (SEM) for normally distributed variables. (Lowercase letters a-b indicate significant differences between the rows with in the same column, * *p* < 0.05, ** *p* < 0.01).

Frozen/Thawed Samples	Aniline Blue–Eosin Staining	CASA Analysis	
	Live Spermatozoa (%)	Total Motility (%)	Progressive Motility (%)
Chrabrany	56.4 ± 1.8 ^a^*	39.2 ± 2.1 ^a^**	20.6 ± 2.6 ^a^**
Hungarian Giant	43.4 ± 3.1 ^b^	20.5 ± 2.0 ^b^	8.5 ± 1.3 ^b^

**Table 4:** Sperm quality of frozen/thawed samples according to flow cytometric measurements in Chrabrany and Hungarian Giant rabbit breeds. Values are presented as mean ± standard error of the mean (SEM) for normally distributed variables and as median (interquartile range, IQR) for the non-normally distributed variable (acrosomal status). IQR represents the 25th–75th percentile range. (Lowercase letters a-b indicate significant differences between the rows with in the same column, * *p* < 0.05, ** *p* < 0.01).

Frozen/Thawed Samples	Plasma Membrane Integrity (%)	Dead/Membrane Compromise (%)	Mitochondrial Activity (%)	Acrosomal Status (%)	ROS (%)
Chrabrany	29.6 ± 1.8	66.4 ± 1.9	30.5 ± 1.7	46.6 (45.2–47.9) ^a^**	24.6 ± 1.1 ^a^*
Hungarian Giant	29.9 ± 2.2	70 ± 2.2	31.4 ± 2.6	70.2 (64.4–77.6) ^b^	33.8 ± 2.5 ^b^

## Data Availability

The raw data supporting the conclusions of this article will be made available by the authors on request.

## References

[1] Mocé E., Vicente J.S. (2009). Rabbit sperm cryopreservation: A review. Anim. Reprod. Sci..

[2] Kubovicova E., Makarevich A.V., Balaži A., Vašiček J., Chrenek P. (2021). Factors affecting rabbit sperm cryopreservation: A mini-review. Zygote.

[3] Domingo P., Olaciregui M., Gonzales N., De Blas I., Gil L. (2019). Comparison of different semen extenders and cryoprotectant agents to enhance cryopreservation of rabbit spermatozoa. Czech J. Anim. Sci..

[4] Grba J., Kuželova L., Makarevich A., Baláži A., Dragin S., Tekiċ D., Chrenek P. (2024). The effect of ellagic acid on rabbit sperm in vitro parameters after cryopreservation. Czech J. Anim. Sci..

[5] Luque P., Kuželova L., Vozaf J., Baláži A., Chrenek P. (2026). Antioxidant astaxanthin enhances cryosurvival and post-thaw functional parameters of rabbit spermatozoa. Cryobiology.

[6] Singh A.K., Kumar A., Bisla A. (2021). Computer-assisted sperm analysis (CASA) in veterinary science: A review. Indian J. Anim. Sci..

[7] Aires V.A., Hinsch K.D., Muller-Schlosser F., Bonger K., Muller-Schlosser S., Hinsch E. (2003). In vitro and in vivo comparison of egg-yolk-based and soya bean-based extenders for cryopreservation of bovine semen. Theriogenology.

[8] Bousseau S., Brillard J.P., Marquant-Le Guienne B., Guérin B., Camus A., Lechat M. (1998). Comparison of bacteriological qualities of various egg yolk sources and the in vitro and in vivo fertilizing potencial of bovine semen frozen in egg yolk or lecithin based diluents. Theriologenology.

[9] Curry M.R., Redding B.J., Watson P.F. (1995). Determination of water permeability coefficient and its activation energy for rabbit spermatozoa. Cryobiology.

[10] Kissné Váradi É., Drobnyák Á., Barna J., Szabó Z., Török É., Végi B. (2026). Preliminary studies for elaborating a semen cryopreservation protocol of the Hungarian Giant rabbit. Anim. Reprod. Sci..

[11] Stranzinger G.F., Maurer R.R., Paufler S.K. (1971). Fertility of frozen rabbit semen. J. Reprod. Fertil..

[12] Rohloff D., Laiblin C. (1976). Deep-freezing of rabbit spermatozoa. Berl. Munch. Tierarztl. Wochenschr..

[13] Weitze K.F., Helleman C., Krause D., Tischner, Pilch J. (1976). Insemination with rabbit semen frozen in plastic straws. Proceedings of the 8th International Congress on Animal Reproduction and Artificial Insemination.

[14] Gotze V.G., Paufler S. (1976). Fertility results and prenatal mortality following artificial insemination with fresh and deep frozen semen in rabbits after normal induction of ovulation and superovulation. Zuchthyg.

[15] Bamba K., Adams C.E. (1990). Freezing rabbit semen by the use of BF5 diluent. Lab. Anim..

[16] Costantini F.F.A. (1989). nel coniglio, sitemi di conservazione dello sperma. Riv. Coniglicoltura.

[17] Castellini C., Battaglini M., Lattaioli P. (1992). Effects of cryoprotectants and freezing on rabbit semen quality. J. Appl. Rabbit Res..

[18] Kuliková B., Baláži A., Supuka P., Chrenek P. (2017). Quality of fresh and frozen-thawed semen from Slovak native rabbit and its storage in the gene bank. Slovak J. Anim. Sci..

[19] Mocé E., Vicente J.S., Lavara R., Viudes-de Castro M.P., López M., Bolet G. (2005). Characteristics of fresh semen from eight rabbit breeds. Reprod. Domest. Anim..

[20] Mocé E., Vicente J.S., Lavara R. (2003). Effect of freezing–thawing protocols on the performance of semen from three rabbit lines after artificial insemination. Theriogenology.

[21] Thurston L.M., Watson P.F., Holt W.V. (2002). Semen cryopreservation: A genetic explanation for species and individual variation?. Cryoletters.

[22] Váradi É., Végi B., Liptói K., Barna J. (2013). Methods for Cryopreservation of Guinea Fowl Sperm. PLoS ONE.

[23] Vašíček J., Baláži A., Svoradová A., Vozaf J., Dujíčková L., Makarevich A.V., Bauer M., Chrenek P. (2022). Comprehensive flow-cytometric quality assessment of ram sperm intended for gene banking using standard and novel fertility biomarkers. Int. J. Mol. Sci..

[24] Kuželová L., Svoradová A., Vašíček J., Dujíčková L., Vozaf J., Berčík J., Halo M., Halo M., Šmondrková M., Chrenek P. (2026). Membrane and mitochondrial antioxidants improve post-thaw functional quality of stallion spermatozoa. Anim. Reprod. Sci..

[25] Reiczigel J., Harnos A., Solymosi N.B. (2007). Nem Statisztikusoknak.

[26] Gonzáles-Ruiz D., Garcia M., Gutiérrez E., Alcántar-Rodríguez A., Medrano A. Effect of the addition of melatonin during the cryopreservation of rabbit semen in Central Mexico. Proceedings of the 13th World Rabbit Congress.

[27] Di Iorio M., Lauriola F., Rusco G., Antenucci E., Schiavitto M., Iaffaldano N. (2024). Cryopreserving Rabbit Semen: Impact of Varying Sperm Concentrations on Quality and the Standardization of Protocol. Vet. Sci..

[28] Mocé E., Lavara R., Vicente J.S. (2005). Influence of the donor male on the fertility of frozen–thawed rabbit sperm after artificial insemination of females of different genotypes. Reprod. Domest. Anim..

[29] Kuželová L., Svoradová A., Baláži A., Vašiček J., Langraf V., Kolesárová A., Sláma P., Chrenek P. (2024). Enhancing of rabbit sperm cryopreservation with antioxidants Mito-Tempo and berberine. Antioxidants.

[30] Chatterjee S., Gagnon C. (2001). Production of reactive oxygen species by spermatozoa undergoing cooling, freezing, and thawing. Mol. Reprod. Dev..

[31] Khan I.M., Cao Z., Liu H., Khan A., Rahman S.U., Khan M.Z., Sathanawongs A., Zhang Y. (2021). Impact of cryopreservation on spermatozoa freeze-thawed traits and relevance OMICS to assess sperm cryo-tolerance in farm animals. Front. Vet. Sci..

[32] Vozaf J., Baláži A., Vašíček J., Váradi É.K., Végi B., Chrenek P. (2026). Artificial insemination success despite reduced post-thaw motility of cryopreserved semen in local rabbit breed: Short communication. Czech J. Anim. Sci..

